# Health Effects of *Psidium guajava* L. Leaves: An Overview of the Last Decade

**DOI:** 10.3390/ijms18040897

**Published:** 2017-04-24

**Authors:** Elixabet Díaz-de-Cerio, Vito Verardo, Ana María Gómez-Caravaca, Alberto Fernández-Gutiérrez, Antonio Segura-Carretero

**Affiliations:** 1 Department of Analytical Chemistry, Faculty of Sciences, University of Granada, Avd. Fuentenueva s/n, 18071 Granada, Spain; ediazdecerio002@correo.ugr.es (E.D.-d.-C.); albertof@ugr.es (A.F.-G.); ansegura@ugr.es (A.S.-C.); 2 Functional Food Research and Development Center, Health Science Technological Park, Avd. Del Conocimiento, Bioregion Building, 18100 Granada, Spain; 3 Department of Nutrition and Food Science, University of Granada, Campus of Cartuja, 18071 Granada, Spain; vitoverardo@ugr.es

**Keywords:** *Psidium guajava* L. (guava) leaves, traditional medicine, in vitro, in vivo, phenolic compounds, pharmacology

## Abstract

Today, there is increasing interest in discovering new bioactive compounds derived from ethnomedicine. Preparations of guava (*Psidium guajava* L.) leaves have traditionally been used to manage several diseases. The pharmacological research in vitro as well as in vivo has been widely used to demonstrate the potential of the extracts from the leaves for the co-treatment of different ailments with high prevalence worldwide, upholding the traditional medicine in cases such as diabetes mellitus, cardiovascular diseases, cancer, and parasitic infections. Moreover, the biological activity has been attributed to the bioactive composition of the leaves, to some specific phytochemical subclasses, or even to individual compounds. Phenolic compounds in guava leaves have been credited with regulating blood-glucose levels. Thus, the aim of the present review was to compile results from in vitro and in vivo studies carried out with guava leaves over the last decade, relating the effects to their clinical applications in order to focus further research for finding individual bioactive compounds. Some food applications (guava tea and supplementary feed for aquaculture) and some clinical, in vitro, and in vivo outcomes are also included.

## 1. Introduction

Ethnomedicine, which refers to the study of traditional medical practice, is an integral part of the culture and the interpretation of health by indigenous populations in many parts of the world [1]. For example, Indian Ayurveda and traditional Chinese medicine are among the most enduring folk medicines still practiced. These systems try to promote health and improve the quality of life, with therapies based on the use of indigenous drugs of natural origin [2]. Given that plants have been widely used as herbal medicines, several approaches are now being carried out to discover new bioactive compounds [3].

**Figure 1:**
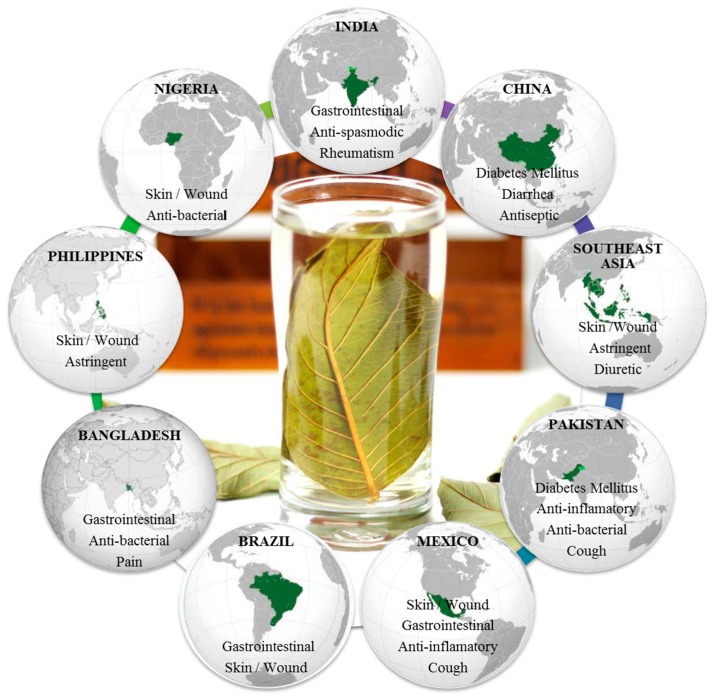
Main traditional uses of guava leaves in the principal producer countries.

*Psidium guajava* L., popularly known as guava, is a small tree belonging to the myrtle family (Myrtaceae) [4]. Native to tropical areas from southern Mexico to northern South America, guava trees have been grown by many other countries having tropical and subtropical climates, thus allowing production around the world [5]. Traditionally, preparations of the leaves have been used in folk medicine in several countries, mainly as anti-diarrheal remedy [6]. Moreover, other several uses have been described elsewhere on all continents, with the exception of Europe [6,7,8]. Figure 1 summarizes the main traditional uses of guava leaves in the main producer countries. Depending upon the illness, the application of the remedy is either oral or topical. The consumption of decoction, infusion, and boiled preparations is the most common way to overcome several disorders, such as rheumatism, diarrhea, diabetes mellitus, and cough, in India, China, Pakistan, and Bangladesh [6,7,8,9], while in Southeast Asia the decoction is used as gargle for mouth ulcers [6,8,9] and as anti-bactericidal in Nigeria [8,9]. For skin and wound applications, poultice is externally used in Mexico, Brazil, Philippines, and Nigeria [6,7,8,9]. In addition, chewing stick is used for oral care in Nigeria [9]. 

Currently, there is increasing interest in studying of plants regarding their chemical components of bioactive compounds, their effects on several diseases, and their use for human health as functional foods and/or nutraceuticals [10]. In recent years, guava leaves tea and some complementary guava products are available in several shops in Japan as well as on the Internet [11], because guava leaf phenolic compounds have been claimed to be food for specified health use (FOSHU), since they have beneficial health effects related to the modulation of blood–sugar level [12]. Thus, the aim of this review was to summarize the biological activities, in vitro and in vivo, studied in the last decade on *P. guajava* L. leaves, relating them to the international classification of diseases provided by the World Health Organization. In addition, the beneficial effects of some applications of guava leaves are also been examined. For this purpose, a comprehensive review of the literature from 2004 to 2016 was done, although more recent studies have also been included. Reviewed journals, websites, books, and several databases as “Scopus”, “Google Scholar”, “PubMed”, and “ScienceDirect”, were used to compile them. To ensure that relevant works are included, terms such as “*Psidium guajava*”, “guava”, “leaves”, “in vitro”, “in vivo”, “clinical”, “trial”, “food application”, and those related with the diseases such as “bacteria”, “cancer”, “blood”, “glycaemia”, and “oral”, among others were matched in the search. Only complete available works published in English, Spanish, and Portuguese have been included.

## 2. Pharmacological Properties

### 2.1. In Vitro Studies

#### 2.1.1. Infectious and Parasitic Diseases

Aqueous and organic extracts of guava leaves have been demonstrated to have antibacterial activity due to an inhibitory effect against antibiotics-resistant clinical isolates of *Staphylococcus aureus* strains [13,14]. Despite using the same diffusion method, differences are noticed in their inhibition zones, as shown in Table 1, probably due to extraction method or the dose assayed. A methanol extract exerted antibacterial effects, preventing the growth of different strains from several bacteria such as *Staphylococcus aureus*, *Escherichia coli*, *Pseudomonas aeruginosa*, *Proteus* spp., and *Shigella* spp. [15]. Furthermore, different extracts of the leaves such as aqueous, acetone–water, methanolic, spray-dried extracts, and the essential oil, showed potential inhibitory activity against Gram-positive and Gram-negative bacteria and fungi [16,17,18,19,20]. In these works, it is noticeable that Gram-positive bacteria exhibited greater inhibition zones and minimum inhibitory concentrations (MICs) than Gram-negative. Concerning the anti-fungal activity, less inhibition than bacteria is reported [16,17], except for *Candida krusei* and *Candida glabrata* which provided higher inhibition [18], and for *Aspergillus* spp. for which no activity was found [16] (Table 1). Moreover, Bezerra et al. [21] evaluated the effect of guava leaves on different bacterial strains, concluding that the synergistic action between the leaves and various antibiotics boosted its anti-bacterial activity. This effect was also observed by Betoni et al. [22] with target drugs for the protein synthesis, cell-wall synthesis, and folic acid. However, the latter did not find synergic effect with gentamicin, perhaps because the time of maceration was lower than the time used by Bezerra et al. [21], and also the solvent was different (Table 1).

Metwally et al. [23] associated the antimicrobial activity against some bacteria and fungi with five flavonoids isolated from the leaves. This effect was also related to the concentration of tannins in the leaves [24] and to the content of gallic acid and catechin [19]. Additionally, the activity against bacterial and fungal pathogens was traced to betulinic acid and lupeol [25]. In fact, these works are focused on the activity of these compounds, rather than on the effect of the whole extract of the leaves.

In addition, leaf acetone extract of *P. guajava* has also exhibited moderate acaricidal and insecticidal activities causing the dead of *Hippobosca maculata* adult fly [26].

Furthermore, Adeyemi et al. [27] suggested that an ethanol extract from the leaves function as a trypanocide agent, since its inhibition of *Trypanosoma brucei brucei* growth proved similar to that of the reference drugs. Kaushik et al. [28] proposed the leaves as an anti-malaria agent, due to their inhibitory activity and the resistance indices. Furthermore, the effect of guava leaf essential oil against toxoplasmosis caused by the growth of *Toxoplasma gondii* were reported [29]. Additionally, guava leaves were proposed for the treatment of diarrhea caused by enteric pathogens, since it showed significant inhibitory activity against *Vibrio cholerae* and *V. parahemolyticus*, *Aeromonas hydrophila*, *Escherichia coli*, *Shigella* spp. and *Salmonella* spp. [30,31,32]. It is suppose that the same plant origin and similar extraction procedure makes that these works show comparable inhibition zones for the bacteria tested [30,31], in contrast to the leaves of India and Bangladesh, where MIC values did not show any concordance [31,32] (Table 1). In addition, a reduction was described for *S. flexneri* and *V. cholera* invasion and for their adherence to the human laryngeal epithelial cells, and for the production of *E. coli* heat labile toxin and cholera toxin, as well as their binding to ganglioside monosialic acid enzyme [33]. Moreover, other studies also demonstrated the antimicrobial effect of some bacteria that cause gastrointestinal disorders by different methods [34,35]. In contrast to previous results [20,31], no inhibition of the hydrodistillation and n-hexane extract was found against *E. coli Salmonella* spp. [31] (Table 1).

Furthermore, guava leaf tea helped control of the growth of influenza viruses, including oseltamivir-resistant strains, via the prevention of viral entry into host cells, probably due to the presence of flavonols [36].

#### 2.1.2. Neoplasms

All the results published regarding anti-cancer properties have been summarized in Table 2.

Kawakami et al. [37] evaluated the anti-proliferative activity of guava leaf extract in human-colon adenocarcinoma cell line (COLO320DMA). These authors found that the extract depressed the proliferation rate due to the presence of quercetin and quercetin glycosides. Moreover, different extracts were tested on three cancer cell lines (cervical cancer (HeLa), breast cancer (MDA-MB-231), and osteosarcoma (MG-63)). The extracts showed no anti-proliferative activity towards HeLa cells, although they displayed activity against MDA-MB-231 and MG-63, the ether extract being the most effective, followed by methanol and water extracts. However, ether and methanol extracts presented a cytotoxic effect on non-malignant cell Madine Darby canine kidney (MDCK) [38]. In contrast, an ethanol extract from the stem and leaves reported significant anti-tumor activity on HeLa and colorectal carcinoma (RKO-AS45-1), whereas its effect was less significant for a lung fibroblast cell line (Wi-26VA4) [39]. This difference could be due to the origin of the leaves, compounds in the steam, or even to the extraction method selected. In this context, an organic guava leaf extract provided molecular evidence of cytotoxic or anti-tumor activity in human breast carcinoma benign cells (MCF-7) and also in murine fibrosarcoma (L929sA) [40]. A fact worthy to comment is that the difference noticed in the cytotoxic effect on MDA-MB-231 cell line might be because the extraction differs [38,40]. Furthermore, the aqueous extract of budding guava leaves displayed an anti-tumor effect against human prostate epithelial (PZ-HPV-7) and carcinoma (DU-145) cells in view of the cell-killing-rate coefficients, as well as anti-angiogenesis and anti-migration activities, respectively [41,42].

Regarding the bioactivity of terpenes from guava, an enriched mixture of guajadial, psidial A, and psiguadial A and B proved anti-proliferative effect for nine human cancer lines: leukemia (K-562), breast (MCF-7), resistant ovarian cancer (NCI/ADR-RES), lung (NCI-H460), melanoma (UACC-62), prostate (PC-3), colon (HT-29), ovarian (OVCAR-3), and kidney (786-0) [43]. The apoptotic effect of β-caryophyllene oxide (CPO) on MCF-7 and PC-3 cell lines was also demonstrated because of its ability to interfere with multiple signaling cascades involved in tumor genesis [44]. Moreover, the essential oil from guava leaves exerted an anti-proliferative effect on human-mouth epidermal carcinoma (KB) and murine leukemia (P388) cell lines [45], while a hexane fraction of the leaves showed a cytotoxic effect against leukemia (Kasumi-1) cancer-cell line at higher half maximal inhibitory concentration (IC_50_), probably due to a less concentration of the bioactive compounds of the leaves [46]. Finally, cytotoxic and apoptotic effect in PC-3 cells and apoptotic effect in LNCaP cells was reported. The lack of cytotoxic effect in LNCaP might be because the cell growth is androgen-dependent, while in PC-3 is androgen-independent. [47]. Comparing these data with those reported by Park et al. [44], high concentration is needed for causing cell death, and a weak effect is found on early apoptotic cell. The main difference between these works is the composition of the extract, so it could be concluded that an antagonist effect is produced amongst the isolated compounds by Ryu et al. [47].

#### 2.1.3. Diseases of the Blood and Immune System

A fermented guava leaf extract was tested in mouse macrophage (RAW 264.7) cells. The results confirmed its potential to decrease the expression of lipopolysaccharide-inducible nitric oxide synthase and cyclooxygenase-2 proteins level, two pro-inflammatory mediators, through the down-regulation of nuclear factor-κB transcriptional activity (NF-κB) [48]. This biological activity was also reported in other works [40,49,50]. Briefly, Jang et al. [49] evaluating the prostaglandin E_2_ production found that the inhibitory effect was highly correlated to the total phenolic content. Kaileh et al. [40] suggested that the suppression of the nuclear factor-κB could be at the transcriptional level because of the lack of binding between nuclear factor-κB and DNA in murine fibrosarcoma (L929sA) and two breast-cancer cell lines (MDA-MB231 and MCF7). At the same time, Jang et al. [50] found that the lipopolysaccharide-induced production of nitric oxide and prostaglandin E_2_ was due to the ability of guava leaf extract to suppress phosphorylation in protein expression. Moreover, Sen et al. [51] verified the inhibition of nuclear factor-κB activation in *Labeo rohita* head-kidney macrophages by the flavonoid fraction of guava leaf extract and Jang et al. [52] improved the inhibition of lipopolysaccharide-induced prostaglandin E_2_ and nitric oxide production by optimizing of the extraction conditions. Furthermore, methanol and ethanol leaf extracts also showed the inhibition of hypotonicity-induced lysis of erythrocyte membrane [53]. Meanwhile, Laily et al. [54] suggested the use of guava leaves as immune-stimulant agent because they modulated the lymphocyte proliferation response.

The results for this activity, confirm the potential of guava leaves as an anti-inflammatory treatment and as immune-system stimulatory agent. As is shown in Table 3, a general trend is reported in every work, although the differences noticed in the data are probably due to the different extraction method and to the doses assayed, or even to the harvesting time of the leaves. However, the mechanism should be further studied since two different pathways are suggested for the down-regulation of NF-κB.

#### 2.1.4. Endocrine and Metabolic Diseases

Several works have focused on elucidating the anti-diabetic compounds present in guava leaves (Table 4). Although the origin of the leaves remains different, the presence of these compounds has demonstrated the hypoglycemic effect of the leaves via different assays. However, the main mode of action seems to be due to an inhibition of the enzymes related to this activity.

The anti-glycative potential of the guava leaves was evaluated, with the conclusion that the extract inhibited, in vitro, the formation of advanced glycation end-products formation [55]. Moreover, the aqueous guava leaf extract, in an albumin/glucose model system, also exerted the same effect and indeed inhibited Amadori products. Gallic acid, catechin and quercetin exhibited over 80% inhibitory effects whereas ferulic acid showed no activity [56]. In another study, seven pure flavonoid compounds (quercetin, kaempferol, guaijaverin, avicularin, myricetin, hyperin, and apigenin) showed strong inhibitory activities against sucrase, maltase, and α-amylase, and a clear synergistic effect against α-glucosidase [57]. Moreover, Deguchi and Miyazaki [58] suggested that the component that inhibited the in vitro activities of α-glucosidase enzymes in guava extract was a polymerized polyphenol. In addition, polysaccharides from guava leaves also exhibited α-glucosidase inhibition [59].

Eidenberger et al. [60] demonstrated the dose-dependent inhibition of guava leaf ethanol extracts on dipeptidyl-peptidase-IV due to the individual flavonol-glycosides: peltatoside, hyperoside, methylquercetin hexoside, isoquercitrin, quercetin/morin pentoside, guaijaverin, and quercetin/morin pentoside. Additionally, the individual flavonol-glycosides found in the guava extract reported no significant differences compared with the uptake of the whole guava extract into epithelial cells (CaCo-2) [60]. In the same cell line, the inhibition of fructose uptake was also tested by Lee et al. [61], who confirmed that catechin and quercetin contributed to the inhibition of glucose transporters. In addition, the enhancement of aqueous guava leaf extract was investigated with regard to glucose uptake in rat clone 9 hepatocytes. Moreover, quercetin was proposed as the active compound responsible for promoting glucose uptake in liver cells and contributing to the alleviation of hypoglycemia in diabetes [62]. Furthermore, Basha and Kumari [63] also estimated the glucose uptake of different extracts. The methanol extract of guava leaves was found to be the most efficient in lowering glucose levels. Basha et al. [64] demonstrated the ability of guavanoic-acid-mediated gold nanoparticles to inhibit the protein tyrosine phosphatase 1B activity.

Indeed, a guava leaf ethanol extract was tested in pre-adipocyte cell line (3T3-L1), which showed its ability to inhibit adipocyte differentiation via down-regulation of adipogenic transcription factors and markers, and hence may prevent obesity in vivo [65]. To evaluate the potential of the leaves on glucose uptake and glycogen synthesis, an aqueous extract was used in insulin-resistant mouse (FL83B) cells. The results confirmed the improved expression and phosphorylation of insulin signaling-related proteins, promoting glycogen synthesis and glycolysis pathways. In fact, this work provides new insights into the mechanisms through which the guava extract improves insulin resistance in the hepatocytes [66]. In the same cell line, vescalagin was postulated as the active component that may alleviate the insulin resistance in mouse hepatocytes [67].

In this sense, the latest study made in L6 myoblasts and myotubes cells confirmed that the glucose uptake recruitment followed a wortmannin-dependent pathway. In addition, guava leaves also inhibited aldose reductase activity, up-regulated gene- and protein-level expression of several insulin receptors and also improved cellular-level glucose uptake [68].

#### 2.1.5. Diseases of the Circulatory System

Cardiovascular disorders have been related to the endothelial cell damage that causes atherosclerosis. In this sense, extracts from budding guava leaves demonstrated a protective, in vitro, effect in bovine aortal endothelial cells, delaying low-density lipoprotein oxidation and preventing oxidized low-density lipoprotein cytotoxicity [69]. A similar effect was also noted in human umbilical-vein endothelial cell due to the ability of saving cell-viability reduction, suppressing reactive oxygen species production and nitric oxide release, as well as inhibiting the expression of NF-κB [70]. Moreover, budding guava leaves also showed their ability as an anticoagulant in plasma, since they reduced thrombin clotting time and inhibited the activity of antithrombin III. Thus, they could help to reduce the development of cardiovascular complications [71].

In addition, flavonoids and phenolic acids in the leaves could contribute to the prevention and amelioration of gout and hypertension, since, in rat-tissues homogenates, they inhibit the activity of two enzymes related to the development of both diseases (xanthine oxidase and angiotensin 1-converting enzymes) [72].

#### 2.1.6. Diseases of the Digestive System

Guaijaverin, isolated from guava leaves, displayed high inhibitory activity against *Streptococcus mutans*. In fact, guaijaverin exhibited its ability as an anti-plaque agent, becoming an alternative for oral care [73]. Furthermore, guava leaves showed greater bactericidal effect on early (*Streptococcus sanguinis*) and late (*S. mutans*) colonizers compared to *Mangifera indica* L. and *Mentha piperita* L. leaves, whereas, when they are compared with the plant extract mixture, the effect is slightly lower. By contrast, guava leaves showed similar and higher anti-adherence effect than the plant mixture [74]. In another study, the whole extract was tested on the cell-surface hydrophobicity of selected early settlers and primary colonizers of dental plaque, showing its ability to alter and disturb the surface characteristics of the agents, making them less adherent [75,76,77], and also delayed in the generation of dental biofilm by targeting growth, adherence, and co-aggregation [78]. This property could be due to the presence of flavonoids and tannins detected in *P. guajava* [79]. Shekar et al. [80] also confirmed the use of the leaves as anti-plaque agents against *Streptococcus mutans*, *S. sanguinis*, and *S. salivarius*. Kwamin et al. [81] discovered the effectiveness of guava leaf extract in the leukotoxin neutralization of *Aggregatibacter actinomycetemcomitans*, leading it to be considered as a possible agent for the treatment of aggressive forms of periodontitis. In addition, extracts rich in guava flavonoids have demonstrated their potential for preventing dental caries due to the growth inhibition of the oral flora [82]. Moreover, its soothing of toothache has been verified based on the analgesic, anti-inflammatory, and anti-microbial activity properties [83] and it has been reviewed positively as an adjutant for treating periodontal disease [84].

Concerning the liver disorders, the cytotoxic and hepato-protective effects of guava leaves were reported. Studies carried out in clone 9 cells treated with different extracts of the leaves showed that only ethanol and acetone extracts tend to have cytotoxicity effect at high concentrations. Moreover, the ethanol extract showed hepato-protective activity, although the hot-water extract reported greater effect and lower cytotoxicity [85].

Table 5 compiles the methodology followed and the results reported in the present works. It is important to keep in mind that the origin, the selection of the extraction method or solvent, and the concentration of the extract tested generally provide different data. For example, comparing data for inhibition zones, best results are noticed at long maceration time in acetone, which seems to be a better extracting solvent than ethanol [77,78,80,82]. Hydrophobicity depends on the origin of the leaves, the extraction method, and the concentration of the extract tested, and it also depends on the lipophilic (index > 70%) or hydrophilic nature of the strain [73,75,79]. Finally, minimum inhibitory concentration relies on all factors.

#### 2.1.7. Diseases of the Skin and Subcutaneous Tissue

Qa’dan et al. [86] described the antimicrobial effect of a leaf extract against the main developer of acne lesions, *Propionibacterium acnes*, and other organisms isolated from acne lesions. The antimicrobial activity was also displayed against pathogenic bacteria associated with wound, skin, and soft-tissue infections [87]. Furthermore, antifungal properties have also been studied by Padrón-Márquez et al. [88]. The acetone and methanol extracts displayed relevant activity against dermatophytic fungi, and thus could be considered as new agents against skin disease. Furthermore, phenols from the leaves were tested on human-skin fibroblast cells and showed antifungal properties [89].

In addition, the tyrosinase inhibitory activities of 4 different parts (branch, fruit, leaf, and seed) of guava, extracted with acetone, ethanol, methanol, and water were tested by You et al. [90] who reported that the ethanol extract from the leaves reached the highest activity. Therefore, the leaves might be appropriate for both boosting the whitening of skin and inhibiting browning. In addition, in a human keratinocyte cell line, an ethyl acetate extract showed a positive effect on atopic dermatitis via the inhibition of cytokine-induced Th2 chemokine expression [91].

Lee et al. [92] carried out the first electrophysiological study based on ultraviolet (UV)-induced melanogenesis with guava leaves. The authors suggested the use of guava leaves for both direct and indirect prevention of skin melanogenesis caused by UV radiation. In fact they demonstrate that methanolic guava leaves extract inhibits tyrosinase, that is the key enzyme in melanin synthesis, and ORAI1 channel that has shown to be associated with UV-induced melanogenesis.

#### 2.1.8. Other Activities Related to Several Diseases

An aqueous guava extract showed its ability to decrease the radiolabeling of blood constituent due to an antioxidant action and/or because it alters the membrane structures involved in ion transport into cells [93]. Guava leaves also have been demonstrated to possess anti-allergic effects in rat mast (RBL-2H3) cell line by the inhibition of degranulation and cytokine production, as well as blocking high-affinity immunoglobulin E-receptor signaling [94].

### 2.2. In Vivo Studies

#### 2.2.1. Infectious and Parasitic Diseases

After checking the effect of guava leaf extract, in vitro, against *Aeromonas hydrophila*, in vivo experiments were carried out in tilapia (*Oreochromis niloticus*), indicating the potential use of *P. guajava* as environmentally friendly antibiotic [95]. The leaves also had anti-viral and anti-bacterial activity towards shrimp (*Penaeus monodon*) pathogens such as yellow-head virus, white spot syndrome virus, and *Vibrio harvey*. In addition, guava leaf extract improved the activities of prophenoloxidase and nitric oxide synthase in serum, and of superoxide dismutase, acid phosphatase, alkaline phosphatase, and lysozyme in serum and hepatopancreas [96]. 

Furthermore, guava leaves have been suggested for managing sleeping sickness, since they exhibited trypanocidal effect in albino rats [97]; the extract ameliorate the tissue-lipid peroxidation associated to trypanosomosis, as well as raising the level of the glutathione concentration [98]. The leaves also showed anti-malarial effect in BALB/c mice infected with *Plasmodium berghei* via parasitemia suppression [99]. Moreover, guava leaves are also recommended for treating infectious diarrhea since they prevented intestinal colonization of *Citrobacter rodentium* in Swiss albino mice [100]. In chicks, guava leaf extract enabled the control of diarrhea produced by *E. coli* and reduced the severity of its symptomatology [101]. In mice, the improvement of cholera symptoms caused by *V. cholerae*, a human pathogen, was also confirmed by Shittu et al. [102].

In addition, anti-helminthic properties towards gastro-intestinal nematodes have been found, as a result of the presence of condensed tannins in the guava plant, which raised the levels of hemoglobin, packed cell volume, total protein, globulin, glucose, and calcium, and lowered the levels of blood urea [103].

All the results published regarding in vivo anti-bacterial properties have been summarized in Table 6.

#### 2.2.2. Neoplasms

Only one study is available on the anti-tumor effect that could be related to the phenolic composition of guava leaves. An ethanol extract of the leaves was administrated to B6 mice after inoculation of melanoma cells. The results suggested that the extract had a vaccine effect, but not a therapeutic effect, against tumors through by depressing T regulatory cells [104].

Moreover, the meroterpene-enriched fraction of guava leaves, containing guajadial, psidial A, and psiguadial A and B, was evaluated in vivo in a solid Ehrlich murine breast-adenocarcinoma model. The results suggested that these compounds may act as phytoestrogens, presenting tissue-specific antagonistic and agonistic activity on estrogen receptors [43]. These data partially confirmed the results in vitro obtained by Ryu et al. [47].

#### 2.2.3. Diseases of the Blood and Immune System

Among blood diseases, anemia indicates a failure in the immune system. In this sense, guava extract presented an anti-anemic effect in trypanosomosis-infected Wistar rats, improving the values of hemoglobin, packed cell volume, red-blood cell counts, mean corpuscular volume, and mean concentration hemoglobin count while decreasing white-blood cell and neutrophil levels [105]. Moreover, the same trend in the hematological analyses was also recorded in mice. After the administration of guava leaf extract, no alterations on the erythron were detected [106]. Nevertheless, results differ because subjects under study are different, also the duration of the treatment, the extraction method and the dose assayed (Table 7).

The anti-inflammatory response of the leaves was dose-dependent in induced hyperalgesia in Sprague-Dawley rats, decreasing in paw-withdrawal latency, and significantly improving the survival rate of mice with lethal endotoxemia [50]. Moreover, the anti-inflammatory activity of aqueous and acetone–water extracts of the leaves was also confirmed in Swiss mice by reducing the amount of leukocyte migration. The acetone–water extract also exhibited peripheral analgesic activity, probably by blocking the effect or the release of endogenous substances that excite pain-nerve endings [19]. The analgesic effect in albino rats was also reported. The ethanol extract reduced the writhing response [107], and a jumping response was found after the administration of a distilled extract (combination of methanol and aqueous extracts) [108]. In this case, the writhing response for both Swiss mice and Wistar rats seems to be comparable, although the dose assayed is completely different (Table 7).

#### 2.2.4. Endocrine and Metabolic Diseases

Guava leaves have shown their potential against one of the diseases with the highest incidence level worldwide, diabetes mellitus, and also towards biochemical changes caused by the disease. In spite of being leaves from different countries, treatments in different subjects or even different data, the same trend is followed in these works (Table 8).

The effect of aqueous guava leaf extract was investigated in rabbits, fed a high-cholesterol diet. Treatment with guava leaves reduced the plasma-cholesterol level, caused a remarkable spike in high-density lipoprotein, a dip in low-density lipoprotein levels, and significantly reduced the associated hyperglycemia. In addition, the extract showed hypolipidemic and hypoglycemic potentials in hypercholesterolemic rabbits [109]. Furthermore, guava leaves reduced oxidative stress induced by hypercholesterolemia in rats [110].

In addition, the anti-diabetic effect was also evaluated in *Lepr^db^*/*Lepr^db^* mice and significant blood-glucose-lowering effects were observed. In addition, histological analysis revealed a significant reduction in the number of lipid droplets, which, furthermore, at least in part, could be mediated via the inhibition of protein tyrosine phosphatase 1B [111]. 

In streptozotocin-induced diabetic rats, the administration of oral doses of aqueous and ethanol extracts from guava leaves could alter the Ca:Mg ratio [112]; however, in low-dose streptozotocin and nicotinamide-induced Sprague-Dawley diabetic rats, long-term administration of guava leaf extracts raised the plasma-insulin level, the glucose utilization, and the activity of hepatic enzymes [113]. Moreover, the leaves also lowered blood glucose levels and decreased protein glycation [55].

In agreement with the above, a lower blood-glucose level was also reported in alloxan-induced diabetic rats. Additionally, no side effects were observed in certain liver enzymes (alkaline phosphatase and aspartate aminotransferase) whereas alanine aminotransferase activity declined [114]. In alloxan-induced diabetic rats, a decrease was also found in blood glucose, total cholesterol, triglycerides, low-density lipoprotein cholesterol, very low-density lipoprotein cholesterol, and a significant increase in high-density lipoprotein cholesterol after 21 days of treatment with guava leaf ethanolic extract [115].

Among the works that evaluated only biochemical parameters, guava leaf extract promoted changes due to an alteration on the activity of alkaline phosphatase, aspartate aminotransferase, alanine aminotransferase, and acid phosphatase in the kidney, liver, and serum [106,116]. In addition, Adeyemi and Akanji [117] evaluated the effect of daily administration of guava leaves, demonstrating the alteration of the serum homeostasis and the pathological variations in rat tissues. 

#### 2.2.5. Diseases of the Circulatory System

Ademiluyi et al. [118] assessed the lipid peroxidation in rats after checking the antihypertensive effect, in vitro, of red and white guava leaves. The work concluded that the activity may be related to rosmarinic acid, eugenol, carvacrol, catechin, and caffeic acid since they were the major constituents of their extracts. In addition, this activity was supported by the biphasic and contractile effect on rat vascular smooth muscles [119,120].

In addition, atherosclerosis development was reduced in apoE-knockout mice by guava leaf extracts. In fact, the effect was connected to the presence of ethyl gallate and quercetin [121,122]. In streptozotocin-induced diabetic rats, vascular reactivity to vasoconstrictor agents was reduced, as was vessel atherosclerosis [112]. Furthermore, Soman et al. [123] found that an ethyl acetate fraction of guava leaves reduced cardiac hypertrophy in streptozotocin-induced diabetic rats due to an anti-glycative effect.

#### 2.2.6. Diseases of the Digestive System

In the digestive system, formed by the gastrointestinal tract plus the group organs necessary for the digestion, guava leaves have demonstrated activity towards different parts.

On the one hand, the leaves have shown the ability to protect the stomach against ulceration by inhibiting gastric lesions, reducing gastric secretory volume, and acid secretion, and raising the gastric pH [124,125,126]. This anti-ulcer activity, resulting from the protection of the mucosa, was related to the flavonoids in the leaves [127]. Despite of the subject employed for the assay, similar data are reported in these works (Table 9). The anti-diarrheal activity of guava leaf aqueous extract was evaluated on experimentally induced diarrhea in rodents. The extract performed in the same way as the control drugs, offering protection, inhibiting intestinal transit, and delaying gastric emptying [128]. Another study attributed this activity to a dual action between the antimicrobial effect and the reduction in gastrointestinal motility ability of the extract [129]. In rabbits, the anti-spasmodic effects were connected to a calcium channel blocking activity, which explains the inhibitory effect on gut motility. The anti-diarrheal protection was also tested in mice [130]. As is shown in Table 9, the anti-diarrheal activity is dose-dependent, although the protection varied depending on the subjects.

On the other hand, guava leaves exhibited hepato-protective effect due to the reduction of serum parameters of hepatic enzymes markers and histopathological alterations in the acute liver damage induced in rats [131,132,133,134,135]. Here, a dose-dependent effect is also found. However, decoction of the leaves seems to be the best option for the extraction of the compounds that exhibited this activity (Table 9).

#### 2.2.7. Diseases of the Skin and Subcutaneous Tissue

Guava leaves have been suggested as a therapeutic agent to control pruritus in atopic dermatitis. The improvement of the skin lesions was due to a reduction in serum immunoglobulin E level and in the eczematous symptoms [136]. Moreover, the epithelium was repaired with connective tissue and absence or moderate presence of inflammatory cells by the leaves. As a result, the leaves exhibited wound healing properties [137]. Furthermore, guava leaf extract was tested on rat skin, and exhibited inhibitory activity towards an active cutaneous anaphylaxis reaction [138].

#### 2.2.8. Other Activities Related to Several Diseases

Triterpenoids from guava leaves were suggested as a potential therapeutic approach for treating diabetic peripheral neuropathy, as they enhanced physical functions and offered neuronal protection towards the suppression of the expression of pro-inflammatory cytokines [139]. In addition, the leaves can act as radio modulators for cancer patients because by preventing DNA damage and apoptosis. [140], as well as protective agents by restoring the normal values of sperm viability, sperm count, sperm motility, and sperm-head abnormality caused by caffeine-induced spermatotoxicity [141].

Moreover, the consumption of guava leaf tea was evaluated, in vivo, in the inhibition of cytochrome P450 (CYP) 3A-mediated drug metabolism by the interaction between guava tea and several drugs [11,142]. Matsuda et al. [11] investigated the consequence of the ingestion of guava tea for two weeks in rats, and the effect with an anxiolytic drug. The short-term consumption of the tea had little effect on the assays performed. This weak influence was due to the absence of interaction between the tea and midazolam in the metabolism studied. In addition, two in vivo studies were made in rats, to evaluate the interaction of guava leaf tea with an anti-coagulant drug (warfarin) [142]. Kaneko et al. [141] suggested that because the tea contained compounds that block the affinity between the enzyme and phenolic compounds of the tea, long-term administration showed a low probability of causing drug-metabolizing enzymes. Moreover, short-term administration revealed that the tea did not interfere with coagulation, meaning that the tea consumption did not alter the pharmacological effect and displayed no side effects.

### 2.3. Clinical Trials

To test the effect of guava leaf extract, several randomized clinical trials have been conducted during the last two decades, although only two studies are available in the last decade. One of the studies consisted of evaluating the effect of guava leaf extract pills on primary dysmenorrhea disorder. For this, 197 women were divided into four groups, and each received a different dosage: 3 and 6 mg extract/day, 300 mg placebo/day and 1200 mg ibuprofen/day. The administration took place over five days during three consecutive cycles. The results demonstrated that 6 mg extract/day alleviated menstrual pain and could replace the use of medicaments like ibuprofen. In fact, guava leaves could be used as a broad-spectrum phyto-drug and not only as an anti-spasmodic agent [143]. Furthermore, Deguchi and Miyazaki [58] reviewed several works regarding the effect of the intake of a commercial guava leaf tea (Bansoureicha^®^, Yakult Honsha, Tokyo, Japan) on different pathologies of diabetes mellitus illness such as the influence on postprandial blood glucose, on insulin resistance and on hypertriglyceridemia and hypercholesterolemia. The authors concluded that the ingestion of guava leaf tea can ameliorate the symptoms of diabetes mellitus and that it could be used as an alimentotherapy.

## 3. Other Applications

Further applications found with guava leaves are listed below: firstly, to prepare gelatin beads with marine-fish gelatin for various applications such as medicine, and the food and pharmaceutical industries [144]. Secondly, Giri et al. [145] suggested guava leaves as supplementary feed for the fish species *Labeo rohita*, due to the immune-stimulatory effect. The same conclusion was reached by Fawole et al. [146] in *L. rohita*. Thirdly, Gobi et al. [147] reported that guava leaf powder, mixed with a commercial diet, strengthened the immunological response of *Oreochromis mossambicus*, and recommended the leaves as feed complement in aquaculture.

## 4. Conclusions

Traditional claims generally require experimental research to establish their effectiveness. In this regard, ethnomedicine applications of *Psidium guajava* L. leaves have been verified by several researches over the last decade against many disorders, demonstrating its potential in the treatment of the most common worldwide diseases. In addition, the effects of the leaves have been related to individual compounds such as quercetin, catechin, vescalagin, gallic acid, peltatoside, hyperoside, isoquercitrin, and guaijaverin.

Future prospects should be aimed at investigating the biodiversity of guava and/or the purification of the different compounds present in guava leaves in order to obtain functional ingredients for further uses as alternative agents in natural therapeutic approaches.

## Figures and Tables

**Table 1:** In vitro assays against infectious and parasitic diseases.

Origin	Extraction Method	Major Constituent	Microorganisms	Assay	Main Results	Ref.
Saudi Arabia	Decoction (30 min)	-	*Staphylococcus aureus* strains	Agar well diffusion assay	At 200 µL: iz ≤ 30 mm.	[13]
India	Soxhlet with MeOH (12 h), maceration in H_2_O (4 h)	-	*S. aureus* strains	Agar well diffusion assay, time-kill of bacterial cell, SDS-PAGE analysis, and cellular toxicity to human erythrocytes assays	At 20 mg/L: iz ≤ 20 mm, MIC: 25 µg/mL (MeOH) and 7.5 mg/mL (H_2_O). MBC: 1.25 and 12.5 mg/mL, respectively, 10 h to kill bacteria, ↑ degradation of protein, no hemolysis.	[14]
Nigeria	Maceration in MeOH (48 h)	-	*S. aureus*, *Escherichia coli*, *Pseudomonas aeruginosa*, *Proteus* spp., and *Shigella* spp.	Agar well diffusion assay	At 20 mg/mL: iz ≤ 18 mm; 81.8% prevention growth.	[15]
India	Maceration with agitation in MeOH, Ac, and DMF (12 h)	-	G-p and G-n bacteria and fungi (91 clinically important strains)	Disc diffusion assay	At 25 mg/mL: against g-p 70% MeOH > 80% Ac > 50% DE, ↓ 76.36% g-n bacteria. Fungi 56% Ac > 38% ME > 31% DMF. No activity against *Citrobacter* spp., *Alcaligenes fecalis*, and *Aspergillus* spp.	[16]
India	Soxhlet with MeOH (4 h)	Phytochemical screening: mainly flavonoid-glycosides and tannins	Bacteria (*Bacillus subtilis*, *S. aureu*s and *E. coli*), and fungi (*Candida albicans* and *Aspergillus niger*)	Paper disc diffusion assay	At 50 μg/mL: iz ≤ 12.6 mm and 10 mm for bacterial and fungi strains, respectively. *E. coli*: MIC 0.78 μg/mL, MBC 50 μg/mL, and MFC 12.5 μg/mL.	[17]
Brazil	Maceration with stirring in EtOH:H_2_O 70% (*v*/*v*) (50 °C, 1 h)	TPC: 25.93 (% m/m, dry base), TFC: 23.48 (mg/g, dry base)	Fungi (*C. albicans*, *Candida krusei*, and *Candida glabrata*), G-p (*S. aureus*) and G-n (*E. coli* and *P. aeruginosa*)	Microdilution assay	MIC = 80–100 µg/mL (*C. krusei*, *C. glabrata* and *S. aureus*) and MBC, MFC ≤ 250–1000 µg/mL (the others).	[18]
Brazil	Turbo-extraction with water or Ac:H_2_O 70% (*v*/*v*) (20 min)	Gallic acid: 0.065 µg/g, Catechin: 1.04 µg/g	G-p strains (*S. aureus*, *Staphylococcus epidermidis*, and *Enterococcus faecalis*) G-n (*E. coli*, *Salmonella enteritidis*, *Shigella flexneri*, and *Klebsiella pneumoniae*)	Agar-diffusion and microdilution assays	At 5 mg/mL: iz ≤ 20 mm, MIC = 39 μg/mL (*S. epidermis*), MIC < 600 μg/mL (the others).	[19]
India	Soxhlet with n-hexane	Methyl 2,6,10-trimethyltridecanoate (28.86%) and Methyl octadecanoate (22.18%)	G-p: *S. aureus*, *Streptoccocus faecalis*, *Bacillus subtillis*, *Lactobacillus* spp., *Enterococcus aerogenes*, *Acinetobacter* spp. G-n: *E. coli*, *Proteus vulgari*, *Enterobacter aerogenes*, *Salmonella typhimurium*, *P. aeruginosa*, and *K. pneumoniae*	Agar well diffusion assay	At 80 µL: iz ≤ 27 mm, MIC = 3–10 µL.	[20]
Brazil	Maceration in EtOH:H_2_O 70% (*v*/*v*) (72 h)	-	*E. coli*, *P. aeruginosa*, and *S. Aureus*	Microdilution assay	Only *S. aureus* (MIC = 256 mg/mL). Synergic effect with ciprofloxacin and gentamicin at 1024 mg/mL.	[21]
Brazil	Maceration in MeOH:H_2_O 70% (*v*/*v*) (48h)	-	*S. aureus* strains	Disc diffusion assay	MIC 90% = 0.52 mg/mL, at 131.75 mg/mL synergic effect with tetracycline, chloramphenicol, erythromycin, vancomycin, oxacillin, cephalothin, ampicillin, cefoxitin, cotrimoxazole.	[22]
Egypt	Maceration in EtOH:H_2_O 50% (*v*/*v*)	Quercetin, avicularin, guajaverin, isoquercitrin, hyperin	*S. aureus*, *E. coli*, *P. aeruginosa*, and *C. albicans*	Agar well diffusion assay	*S. aureus*: ↑ iz quercetin (28 mm), MIC (μg/mL) guajaverin (0.09–0.19) < avicularin (0.09–0.38) < quercetin (1.25) for all the microorganism tested.	[23]
Indonesia	Maceration in EtOH:H_2_O 30% (*v*/*v*) (3 days)	Tannins (2.35 mg/g)	*E. coli*, *P. aureginosa*, *S. aureus*, *A. niger* and *C. Albicans*	Paper disc diffusion method	iz ≤ 15 mm.	[24]
India	Soxhlet with toluene (72 h)	Betulinic acid and lupeol	Fungi: *Calletotricheme camellie*, *Fussarium equisitae*, *Alterneria alternate*, *Curvularia eragrostidies*, and *Colletrichum Gleosproides*. Bacteria: *E*. *Coli*, *B. Subtillis*, *S. aureus*, and *Enterobactor*	Slide germination method	Bacteria: MIC < 100–200 μg/mL, fungi: MIC < 2.5–10 μg/mL.	[25]
India	Soxhlet with Ac (8 h)	-	*H. bispinosa* Neumann (Acarina: *Ixodidae*) and *H. maculata* Leach (Diptera: *Hippoboscidae*)	Antiparasitic activity method of FAO (2004)	At 3 mg/mL: mortality 100% *H. maculate*, 78% *H. bispinosa*, parasite dead *H. maculata* (LC_50_ = 0.646 mg/mL).	[26]
Nigeria	Maceration with agitation in EtOH:H_2_O 20% and 80% (*v*/*v*) (24 h)	-	*Trypanosoma brucei brucei* and HEK293	Alamar Blue assays	At 238.10 μg/mL: IC_50_ (*T. b. brucei*) = 6.3 μg/mL and 48.9 μg/mL for 80% and 20% extracts, respectively, IC_50_ (HEK293) 30.1 and 24.16%, respectively.	[27]
India	Soxhlet with ethyl acetate and MeOH (8 h)	-	*Plasmodium falciparum* strains	SYBR green assay	IC_50_ 9–18 μg/mL, resistance indices = 0.6 and 1.4 in MeOH and ethyl acetate, respectively.	[28]
Malaysia	Hydrodistillation (3 h)	-	*Toxoplasma gondii*	MTT assay with Vero cells	At 200 µg/mL: No cytotoxic effect (EC_50_ = 37.54 µg/mL), anti-parasitic activity (EC_50_ of 3.94 µg/mL).	[29]
India	Soxhlet with EtOH, and maceration in H_2_O (6 days)	-	*E. coli*, *Shigella* spp., *Salmonella* spp., *Aeromonas* spp., *S. aureus*, and *Candida* spp.	Agar well diffusion assay	H_2_O: iz ≤ 30 mm (max *C. albicans*). EtOH: iz ≤ 31 mm (max *Aeromonas hydrophila*).	[30]
India	Soxhlet with EtOH:H_2_O 70% (*v*/*v*), MeOH, ethyl acetate, and H_2_O	Phytochemical analysis: tannins, saponins, flavonoids, terpenoids, sugars	*E. coli*, *Salmonella* spp., and *Vibrio cholerae*	Agar well diffusion assay	At 1000 µg/mL: iz ≤ 30 mm. MeOH: MIC (100%) > 250 µg/mL. EtOH:H_2_O:MICs (38–65%) > 500 µg/mL and > 750 µg/mL. Ethyl acetate and H_2_O: MICs > 750 µg/mL.	[31]
Bangladesh	Maceration in H_2_O and MeOH:H_2_O 75% (*v*/*v*) (48 h)	-	*V. cholera*	Agar well diffusion assay	MICs = 1250 µg/mL (H_2_O), 850 µg/mL for (MeOH:H_2_O). Antibacterial resistance to trimethoprim/sulfomethoxazole, furazolidone, tetracycline, and erythromycin.	[32]
India	Decoction	Major component: quercetin (2 mg/g)	*E. coli* (heat labile (HLT) and cholera toxin (CT)), *V. cholerae*, *Shigella flexneri*	Microtitre plate based assay. Assays for bacterial colonization (adherence and invasion) and enterotoxins	At 2.7 mg/mL: (EC_50_ = 0.98 (*S. flexneri*) and 2.88% (*V. cholerae*). ↓ adherence and invasion to epithelial cells (EC_50_ = 0.37–1.25% and 0.04–0.25%, respectively). The effect on adherence is not due to quercetin and the invasion is lower than with the extract. ↓ Production of HLT and CT (EC_50_ = 1.03 and 2.69%) and binding to glioside monosialic acid enzyme (EC_50_ = 0.06 and 2.51%).	[33]
Brazil	Soxhlet with n-hexane, ethyl acetate, MeOH, H_2_O (24 h)	-	*S. aureus*, *Salmonella* spp., and *E. coli*	Disc diffusion method	At 1938 µg/disc: iz = 7.00–11.25 mm (Soxhlet), and 11–18 mm (H_2_O). No inhibition to *E. coli* (H_2_O) and *Salmonella* spp. (hexane and ethyl acetate).	[34]
Nigeria	Soxhlet with EtOH:H_2_O 60% (*v*/*v*) (5 h), and H_2_O (3 h)	-	*E. coli* and *S. aureus*	Agar well diffusion assay	At 10 mg/mL: H_2_O: iz = 9–16 mm and 8–11 mm, MICs = 5 and 2.5 mg/mL (*E. coli* and *S. aureus*, respectively). EtOH:H_2_O: iz 12–21 and 11–14 mm, MICs = 1.25 and 0.625 mg/mL, respectively.	[35]
Japan	Infusion (8 min)	Tannin content: 1.11 mg/mL	H1N1 virus strains	19-h Influenza growth inhibition assay	At 0.4 mg/mL: inhibition growth (IC_50_ = 0.05–0.42%).	[36]

Acetone (Ac); *N*,*N*-dimethylformamide (DMF); dodecyl sulfate-polyacrylamide gel electrophoresis (SDS-PAGE); effective concentration (EC_50_); inhibition zone (iz); inhibitory concentration (IC_50_); lethal concentration (LC_50_); minimum bactericidal concentration (MBC); minimum fungicidal concentration (MFC); minimum inhibitory concentration (MIC); total flavonoid content (TFC); total phenolic content (TPC); Tetrazolium (MTT); ↑ increases the affect; ↓ decreases the effect.

**Table 2:** In vitro studies against neoplasm.

Origin	Extraction Method	Major Constituent	Cell Line	Assay	Main Results	Ref.
Japan	Maceration in EtOH:H_2_O 50% (*v*/*v*)	TPC: 71 g/100 g	Human colon adenocarcinoma (COLO320DMA)	Cyclooxygenase and cell proliferation assays	At 1 mg/mL: ↓ human cyclooxygenase activity (IC_50_ 55 and 560 µg/mL PGHS-1 and 2, respectively), ↓ IC_50_ 5.1 µg/mL (PGSH) and 4.5 µg/mL (cyclooxygenase).	[37]
					At 100 µg/mL: Quercetin ↓ IC_50_ = 5.3 (PGSH-1) and 250 µg/mL (PGSH-2), ↓ DNA synthesis rate.	
Malaysia	Soxhlet with ether, MeOH, and H_2_O	-	Cervical cancer (HeLa), breast cancer (MDA-MB-231) and osteosarcoma (MG-63). Control: non-malignant Madin-Darby canine kidney (MDCK)	Methylene blue assay	At 10 mg/mL: HeLa: No anti-proliferative activity.	[38]
					MDA-MB-231: IC_50_ ether extract (4.2 µg/mL) > MeOH (18.6 µg/mL) > H_2_O (55.7 µg/mL).	
					MG-63: same order (IC_50_ of 5.42, 23.25, and 61.88 µg/mL, respectively).	
					MDCK: cytotoxic effect of ether and MeOH extract (IC_50_ = 5.03 and 11.55 µg/mL, respectively).	
Brazil	Maceration in EtOH	TPC: 766.08 mg/g, TFC: 118.90 mg/g	HeLa, colorectal carcinoma (RKO-AS45-1), and lung fibroblast (Wi-26VA4)	MTT assay	At 1 mg/mL: IC_50_ = 15.6 µg/mL (HeLa), 21.2 (RKO) µg/mL, and 68.9 µg/mL (Wi-26VA4).	[39]
Palestine	Maceration in DCM:MeOH 50% (*v*/*v*) (24 h)	-	Murine fibrosarcoma (L929sA), and human breast cancer (MDA-MB-231 and MCF-7)	MTT assay	IC_50_ = 55 µg/mL (L929sA), 820 µg/mL (MCF7 cells), no cytotoxic effect on MDA-MB-231 cells.	[40]
Taiwan	Decoction (30 min)	-	Human prostate carcinoma (DU-145)	MTT, ELISA, gelatinolytic zymography, wound scratch, and chicken chorioallantoic membrane assays	At 0.25 mg/mL: cell suppression (IC_50_ 0.57 mg/mL). ↓ Expressions of VEGF (76.9%), IL-6 (98.8%) and IL-8 (98%), and MMP-2 (100%) and MMP-9 (100%). Suppressed the cell migration (30.9%) and the angiogenesis.	[41]
Taiwan	Decoction (1 h)	TPC: 470.0 mg/g Individual compounds: gallic acid (348), catechin (102), epicatechin (60), rutin (100), quercetin (102), and rutin (100) in mg/g	Human prostate epithelial (PZ-HPV-7) and DU-145	MTT assay	At 1 mg/mL: 100% suppression DU-145 cells. PZ-HPV-7 cells followed an auto-decaying process. Cell-killing rate coefficient (kapp) = 0.03 × 10^3^ phenolic compounds cells/mg h.	[42]
Brazil	Soxhlet with DCM. Maceration with EtOH	Guajadial, psidial A, and psiguajadial A and B	Leukemia (K-562), MCF-7, ovarian cancer (NCI/ADR-RES), lung (NCI-H460), melanoma (UACC-62), prostate (PC-3), colon (HT-29), ovarian (OVCAR-3), and kidney (786-0)	Protocol established by NCI (ELISA test)	At 250 µg/mL: Anti-proliferative activity DCM > EtOH, inhibition growths: 26 (OVCAR-3)-65 (UACC-62) µg/mL due to the major compounds.	[43]
Japan	Maceration with sonication in MeOH:H_2_O 80% (*v*/*v*) (3 h) and isolation	CPO	PC-3 and MCF-7	MTT, annexin V antibody, TUNEL, and western blot assays	At 50 µg/mL: ↓ cell proliferation, ↑ early and late apoptotic effect, down-regulation of PI3K/AKT/mTOR/S6K1 pathway, up-regulation of MAPKs, JNK, ERKs, and p38 MAPK.	[44]
Thailand	Hydrodistillation		Human mouth epidermal carcinoma (KB) and murine leukemia (P388)	MTT assay	At 0.15 mg/mL: KB: 75% cytotoxic effect, IC_50_ = 0.04 mg/mL; At 0.08 mg/mL: P388: 80% cytotoxic effect, IC_50_ = 0.05 mg/mL.	[45]
Jamaica	Maceration in hexane (4 days)	-	Leukemia (Kasumi-1)	MTT assay	IC_50_ = 200 µg/mL.	[46]
Japan	Maceration with sonication in MeOH:H_2_O 80% (*v*/*v*) (3 h). Fractionation with hexane	60 compounds (in hexane fraction): β-eudesmol (11.98%), α-copaene (7.97%), phytol (7.95%), α-patchoulene (3.76%), and CPO (3.63%)	Human prostate cancer (PC-3 and LNCaP)	MTT, annexin V antibody, TUNEL, and western blot assays	At 150 µg/mL: PC-3: ↑ apoptotic effect of the hexane fraction (15%), ↓ effect on early apoptotic cells, ↑ effect for late apoptosis, via the suppression of PI3K/AKT/mTOR/S6K1 and MAPK signalling cascades in both cell lines.	[47]

β-Caryophyllene oxide (CPO); c-jun NH2-terminal kinases (JNK); dichloromethane (DCM); inhibitory concentration (IC_50_); mammalian target of rapamycin (mTOR); mitogen-activated protein kinases (MAPKs); phosphatidylinositol 3-kinase (PI3K); prostaglandin endoperoxide H synthase (PGHS); protein kinase B (AKT); ribosomal protein S6 kinase beta-1 (S6K1); signal-related kinases (ERKs); tetrazolium (MTT); total flavonoid content (TFC); total phenolic content (TPC); ↑ increases the affect; ↓ decreases the effect.

**Table 3:** In vitro assays against diseases of the blood and immune system.

Origin	Extraction Method	Major Constituent	Cells	Assay	Main Results	Ref.
Korea	Maceration in MeOH:H_2_O 70% (*v*/*v*) (5 days)	-	LPS-stimulated RAW 264.7 (Mouse macrophage)	Griess, MTT, ELISA kit, western blot, transient transfection, and luciferase assays	At 125 µg/mL: no cytotoxic effect, ↑ 44–62% inhibition rates. ↓ LPS-induced NO and PEG_2_ ↓ iNOS and COX-2 (↓ I-κBα degradation, ↓ activation NF-κB).	[48]
Palestine	Maceration in DCM:MeOH 50% (*v*/*v*) (24 h)	-	L929sA fibroblast	Transfection and luciferase assays	At 62.5 µg/mL: ↓ expression of IL-6 and NF-κB luciferase reporter gene construct via the NF-κB transactivation level, since no ↓ inhibition of NF-κB/DNA binding.	[40]
Korea	Extraction in MeOH:H_2_O 70% (*v*/*v*) (6 h)	TPC: 426.84 mg (GAE)/g	LPS-stimulated RAW 264.7	MTT, Griess, and ELISA test assays	At 30 µg/mL: no cytotoxic effect. ↓ LPS-induced NO (52.58%) and the production of PGE_2_ (43.45).	[49]
Korea	Extraction in EtOH:H_2_O 55% (*v*/*v*) (4.9 h, 47 °C)	Gallic acid (0.2) and catechin (4.4) in mg/g	LPS-stimulated RAW 264.7	MTT, Griess, ELISA test, RT-PCR, and total western blot assays	At 50 µg/mL: no cytotoxic effect. ↓ LPS-induced NO (>65%) by ↓ iNOS, ↓ PGE_2_ (to basal level) via ↓ COX-2 mRNA. ↓ IL-6. ↓ iNOS and COX-2 due to the down-regulation of ERK1/2 pathway, because no effect was found to other proteins at the dose tested.	[50]
India	Maceration in MeOH:H_2_O 90% (*v*/*v*) (x3)	-	LPS-stimulated in Labeo rohita head-kidney macrophages	MTT, Greiss, ELISA, RT-PCR, and western blot assays	At 200 µg/mL, ↓ LPS-induced NO (75%) by ↓ iNOS-mRNA, ↓ PGE2 (45%) via ↓ production COX-2-mRNA, TNF-α, IL-1β, IL-10, and mRNA expression. Suppressed phosphorylation of MAPK (↓ I-κBα degradation ↓ activation NF-κB).	[51]
Korea	Soxhlet with EtOH:H_2_O 55% (*v*/*v*) (4.9 h, 47 °C)	Gallic acid (0.09) and catechin (0.72) in mg/g	LPS-stimulated RAW 264.7	MTT, Greiss and ELISA test assays	At 30 µg/mL: no cytotoxic effect. ↓ LPS-induced NO (47.5%) and PGE_2_ (45.8).	[52]
India	Maceration with agitation in MeOH and EtOH (24 h)	-	Human blood	HRBC membrane stabilization method	At 200 µg/mL: ↑ 13.8–14.4% prevention of lysis of the membrane.	[53]
Indonesia	Maceration with agitation in EtOH:H_2_O 96% (*v*/*v*) (6 h)	TPC: 101.93 mg GAE/g	Human lymphocyte	MTT assay	0.5 μg/mL: Stimulation index 1.54%.	[54]

Cyclooxygenase-2 (COX-2); dichloromethane (DCM); gallic acid equivalent (GAE); human red blood cell (HRBC); inducible nitric oxide synthase (iNOS); inhibitor of kappa B (I-κBα); interleukin-1β (I-1β); lipopolysaccharide (LPS); mitogen-activated protein kinases (MAPKs); nitric oxide (NO); prostaglandin E_2_ (PEG_2_); reverse transcription-polymerase chain reaction RT-PCR; tetrazolium (MTT); total phenolic content (TPC); transcriptional nuclear factor-κB (NF-κB); Tumor necrosis factor alpha (TNF-α); ↑ increases the affect; ↓ decreases the effect.

**Table 4:** Compounds in guava leaves with anti-diabetic properties in in vitro assays.

Origin	Compound	Assay	Main Results	Ref.
India	Ethyl acetate fraction	In vitro glycation of BSA-fluorescence measurement	In vitro AGEs formation with IC_50_ of 38.95 ± 3.08 μg/mL.	[55]
Taiwan	Gallic acid, catechin and quercetin	In vitro glycation of BSA-fluorescence measurement; Fructosamine assay and Girard-T assay	At 100 μg/mL: 80% inhibitory effects on the formation of α-dicarbonyl compounds at a concentration of 50 µg/mL, inhibitory effects on AGEs formation in BSA glycation systems.	[56]
China	Quercetin, kaempferol, myricetin	Rat intestinal sucrase and maltase inhibitory activities; Porcine pancreatic α-amylase inhibitory activity	At 1.5 mg/mL: inhibitory activities with IC_50_ values of 3.5, 5.2 and 3.0 mM against sucrase, with IC_50_ values of 4.8, 5.6 and 4.1 mM against maltase and with IC_50_ values of 4.8, 5.3 and 4.3 mM against α-amylase, respectively. Synergistic effect against α-glucosidase.	[57]
China	Water-soluble polysaccharides, including GP90 and P90	α-Glucosidase inhibition assay	α-Glucosidase inhibition activity with an EC_50_ of 2.27 µg/mL and 0.18 mg/mL.	[59]
-	Peltatoside, hyperoside, isoquercitrin, guaijaverin and flavonol-glycosides	Spectrophotometric assay; absorption assay into CaCo-2 cells	Concentration of the compounds (0.01 to 0.06 µmol/mL). Individual flavonol-glycosides inhibited DP-IV dose-dependently. The ethanolic guava leaves extract (380 µg/mL) showed a dose-dependent inhibition of DP-IV, with an IC_50_ of 380 μg/mL test assay solution; the highest uptake was from Guaijaverin.	[60]
Korea	Quercetin and catechin	Fructose transport in CaCo-2 cell systems	At 1 mg/mL: inhibition of fructose uptake (55%). At 30 µg/mL: quercetin contributed to both, GLUT2 and 5 transporters, and catechin to GLUT5-mediated fructose uptake inhibition.	[61]
India	Guavanoic acid	Spectrophotometric assay	At 27 μg/mL: remarkable PTP1B inhibitory activity (90%) and in vitro stability in various physiological medium including saline, histidine, cysteine, BSA, HSA and buffers (pH 5, 7 and 9). IC_50_ = 1.14 μg/mL.	[64]
India	n-Hexane, methanol, ethanol and aqueous leaf extracts	Inhibitory glucose diffusion	At 50 g/L: the methanol extract was the most potent with the lowest mean glucose concentration of 201 ± 1.69 mg/dL at the end of 27 h (↓ 93% uptake).	[63]
Japan	70% Ethanol extract	Oil Red O Assay; Real-Time RT	At 100 μg/mL: inhibition of 3T3-L1 differentiation via down-regulation of adipogenic transcription factors and markers (mRNA levels of PPAR-γ, C/EBP-α, and aP2), and suppression of mitotic clonal expansion (at day 4 and 8).	[65]
Taiwan	Aqueous extract	Glucose uptake test; bicinchonic acid method; Western-blot analysis	At 400 µg/mL: ↑ IR (25.1%), p-IR (46.2%), p-IRS (51.2%), PI3K (32.2%), Akt (46.1%), p-Akt (36.3%), GLUT-2 (46.8%), and total glycogen synthase (45.5%).	[66]
Taiwan	Vescalagin	Glucose-uptake test	At 100 µg/mL: Enhancement of glucose uptake in TNF-α-induced insulin-resistant.	[67]

Advanced glycation end products (AGEs); bovine serum albumin (BSA), dipeptidyl peptidase (DP); effective concentration (EC_50_); glucose transporter 2 and 5 (GLUT-2; GLUT-5); human serum albumin (HSA); inhibitory concentration (IC_50_); insulin receptor (IR); insulin receptor substrate (p-IRS (Tyr)); p85 regulatory subunit of phospho-inositide 3 kinase (PI3K (p85)); phosphorylation of the insulin receptor (p-IR (Tyr)); protein kinase B (p-Akt (Ser)); tumor necrosis factor (TNF); ↓ decreases the effect.

**Table 5:** In vitro assays against diseases related to the digestive system.

Origin	Extraction Method	Microorganism(s)/Cells	Assay	Main Results	Ref.
India	Soxhlet with MeOH (4.5 h)	*S. mutans* strains	Agar well diffusion assay, effect on acid production, on sucrose-dependent adherence to smooth glass surfaces, and on sucrose-induced cellular aggregation, and MATH assays	MIC > 5 mg/mL (MeOH). MIC = 2–4 mg/mL (guaijaverin) At sub-MIC (0.125–2 mg/mL): ↑ pH (5 to 6–7), hydrophobicity indexes (3.2–72%), ↓ sucrose-dependent adherence (34–84%) and aggregation.	[73]
Malaysia	Decoction	*S. sanguinis* and *S. mutans*	NAM model system	At 60.95 mg/mL: MIC = 7.62 (*S. sanguinis*) and 3.81(*S. mutans*.) mg/mL. MBC values = 15.24 and 30.48 mg/mL, respectively. At 0.5 mg/mL: ↓ adherence 57 and 60% (single-species) and 88–89% (dual-species).	[74]
Malaysia	Sonication with H_2_O (10 min)	*S. sanguinis*, *S. mitis*, and *Actinomyces* spp.	MATH assay	At 1 mg/mL: ↓ 54.1%, 49.9% and 40.6%, respectively, cell-surface hydrophobicity. At 20 mg/mL: was 64.7, 60.5, and 55.5%, respectively.	[75]
Malaysia	Decoction	*S. sanguinis*, *S. mitis*, and *Actinomyces* spp.	Bacterial growth and generation time rates determinations	At 4 mg/mL: Time growth = 1.22 (*S. sanguinis*, *Actinomyces* spp) and 2.06 h (*S. mitis*) ↓ growth 42.6%, 51.2% and 55%.	[76]
India	Maceration with stirring in EtOH (2 days)	*S. mutans*, *S. sanguinis*, and *S. salivarius*	Agar well diffusion assay	At 10 mg/mL: inhibition zones of 21.17, 18.58, and 23.00 mm, respectively.	[77]
India	Maceration (2 days) and Soxhlet (6 h) with EtOH, H_2_O, and EtOH:H_2_O 50% (*v*/*v*)	*S. mutans* and *S. mitis*	Agar well diffusion assay, sucrose-dependent adherence and cellular co-aggregation activities, and biofilm formation sterile acrylic tooth determinations	At 15 mg/mL: inhibition zone for H_2_O (11.8 mm) to EtOH:H_2_O (25 mm), both by Soxhlet. MIC = 1 mg/mL. EtOH:H_2_O extract: at >0.05 mg/mL: ↓ adherence and co-aggregation, at MIC, ↓ the viable count of dental biofilm (3.50 log_10_ CFU/mL).	[78]
India	Soxhlet with EtOH:H_2_O 50% (*v*/*v*) (6 h)	*S. mutans* and *S. mitis*	MATH assay	At >1 mg/mL ↓ hydrophobicity (index < 40%).	[79]
India	Maceration with stirring (2 days) and Soxhlet with EtOH	*S. mutans*, *S. sanguinis*, and *S. salivarius*	Agar well diffusion assay	At 10 mg/mL: ↑ inhibition zones for maceration extracts (19–23 mm).	[80]
Ghana	Maceration with agitation in EtOH:H_2_O 70% (*v*/*v*) (24 h)	*Aggregatibacter actinomycetemcomitans* strains	Agar well diffusion assay, release of the cytosol enzyme lactate dehydrogenase, fluorescence assisted cell sorter, and ELISA assays	No growth inhibitory effect, although neutralized the cell death and pro-inflammatory response, and restored the morphological alterations induced by the leukotoxin. These effects were due to the direct binding of guava compounds and the leukotoxin.	[81]
India	Maceration in Ac, EtOH, chloroform, MeOH and H_2_O (15 days at 22 °C)	*Neisseria catarrhalis*, *S. mutans*, *S. salivarius*, *Streptococcus viridans*, *Bacillus megaterium*, and *P. aeruginosa*	Agar well diffusion assay	↑ Inhibition zones in Ac (15–29 mm), except for *N. catarrhalis* (20 mm in MeOH).	[82]
India	Maceration in MeOH (72 h). Fractionation with ethyl acetate	*S. aureus* and *S. mutans*	HRBC membrane stabilization method, disc and agar well diffusion assays	MeOH and ethyl acetate fraction ↑ protection (84–99%) to the inflammatory response. Inhibition zones (25–100 µg/mL) = 10.5 to 22 mm by both methods. MICs = 0.48 (ethyl acetate) and 0.62 (MeOH) mg/mL.	[83]
Taiwan	Maceration EtOH, Ac, H_2_O (room temperature and 60 °C) (24 h)	Clone 9 rat liver cells	WST-1 and ALT assays	At >500 μg/mL cytotoxic effect of EtOH and Ac and 600 μg/mL for H_2_O. At <200 μg/mL normal values were observed for H_2_O and Ac, and EtOH (<500 μg/mL). At <100 μg/mL: Hepato-protective effect in EtOH and H_2_O (full range).	[85]

Alanin aminotransferase (ALT); colony forming unit (CFU); human red blood cell (HRBC); microbial adhesion to hydrocarbon test (MATH); minimum bactericidal concentration (MBC); minimum fungicidal concentration (MFC); minimum inhibitory concentration (MIC); nordini’s artificial mouth (NAM); Tetrazolium (WST-1); ↑ increases the affect; ↓ decreases the effect.

**Table 6:** In vivo anti-bacterial effect.

Origin	Extraction Method	Subject	Treatment	Main Results	Ref.
Thailand	Maceration in H_2_O, EtOH, and ether (24 h)	*Oreochromis niloticus*	*Aeromonas hydrophila*	LD_50_ = 3.44 × 106 CFU/mL. ↓ Mortality of the subjects.	[95]
China	-	*Penaeus monodon*	Yellow-head virus, white spot syndrome virus, and *Vibrio harveyi*	Survival rate = 80–95% (↑ Weight (2 to 6 g)). In serum (↑ feed): ↓ PO (7.50 U/mL) and SOD (178.33 U/mL), ↑ NOS (64.80 U/mL). In hepato-pancreas: ↑ SOD (57.32 U/mg), ACP (23.28 U/mg), AKP (19.35 U/mg), and LSZ (3459.946 U/mg).	[96]
Nigeria	Maceration with agitation in EtOH:H_2_O 80% (*v*/*v*) (24 h)	Albino rats	*T. b. brucei*	At 300 mg/kg: ↓ parasitemia; ↑ survival in 24 days.	[97]
Nigeria	Maceration with agitation in EtOH:H_2_O 80% (*v*/*v*) (24 h)	Albino rats	*T. b. brucei*	Administration 1–7 days. ↑ GSH: liver (5.4 to 8.1), kidney (3.3 to 6.0), and serum (0.8 to 2.4), restored in kidney and serum. In the brain, no effect was found. ↓ MDA: serum (13.9 to 5.9), brain (42.8 to 18.1), kidney (27.3 to 17.6), and liver (38.2 to 19.2).	[98]
India	Decoction of the leaves (10 min)	BALB/c mice	*Plasmodium berghei*	At 350 and 1000 mg/kg ↓ parasitemia (73.7% and 85.8%); ↑ survival 15 and 18 days.	[99]
India	Extraction in EtOH:H_2_O 50% (*v*/*v*)	Swiss mice	*Citrobacter rodentium*	At 300 mg/kg: ↓ infection (day 4) of the treatment, and no infection at day 19 (control group at day 24).	[100]
Nigeria	Hidrodistillation and fractionation with ethyl acetate	ISA brown male chicks	*E. coli*	At 100 mg/kg: In 10 days ↓ signs of villous collapse (stunting, matting and fusion of villi), number of wet droppings (12-6); ↑ activity, weight gaining, and feed intake (from 27 to 45 g) in contrast to the infected ones (from 30 to 18 g); ↓ bacterial shedding load (from 60 to 45 CFU/mL).	[101]
Nigeria	Decoction of the leaves	Adult mice	*V. cholera*	At 250 mg/kg: Histopathological observations: mild degenerative, secretory, and inflammatory changes with goblet cells and with most of the exudate (neutrophils and lymphocytes).	[102]
India	-	Adult male goat	*Haemonchus contortus*	90 Days feeding: ↑ Hb (7.2 to 8.6 g/dL), PCV (20.2 to 29.3%), total protein (4.8 to 6.3 g/dL), GLO (2.3 to 3.8 g/dL) (↑ control (2.8)), glucose (43.9 to 52.6 g/dL), and calcium (8.7 to 9.6 mg/dL); ↓ blood urea (47.9 to 29.8 mg/dL) (↓ control (41)). Phosphorus balance, serum albumin levels and serum enzyme activity did not show variation.	[103]

Acid phosphatase (ACP); alkaline phosphatase (AKP); colony forming unit (CFU); globulin (GLO); glutathione (GSH); hemoglobin (Hb); lysozyme (LSZ); malondialdehyde (MDA); median lethal dose (LD_50_); nitric oxide synthase (NOS); packed cell volume (PCV); prophenoloxidase (PO); superoxide dismutase (SOD); ↑ increases the affect; ↓ decreases the effect.

**Table 7:** In vivo studies against diseases of the blood and immune system.

Origin	Extraction Method	Subject	Treatment	Main Results	Ref.
Nigeria	Maceration with agitation in EtOH:H_2_O 80% (*v*/*v*) (24 h)	Wistar rats	*T. b. brucei*/no infected	Treatment (1–7 days) at 150 mg/kg: ↑ Hb (6.5 to 10.7 g/dL), PCV (28.6 to 34.4%), RBCC (4.1 to 5.0 × 10^12^/L), MCV (53.6 to 64.3 fL), and MCHC (21.4 to 31.4 g/dL); ↓ WBC (23.2 to 19.4 × 10^9^/dL) and neutrophil levels (28.9 to 27.3 × 10^3^/mL).Compared to no infected subjects: similar values that obtained in treated-infected animals but with opposite conclusions.	[105]
Nigeria	Extraction in chloroform (24 h)	Mice	No infected	Treatment (28 days) at 45.9 mg/mL: no differences in Hb (12 to 11 g/dL), PCV (37 to 35%), RBCC (6.1 to 5.1 × 10^6^/L), and MCHC (33 to 32 g/dL), and neutrophil levels (13 to 12%); ↑ lymphocyte levels (85 to 92%) and MCV (61 to 69 fL).	[106]
Korea	Extraction in EtOH:H_2_O 55% (*v*/*v*) (4.9 h, 47 °C)	Sprague-Dawley rats and mice	Freund’s complete adjuvant-induced hyperalgesia/LPS-induced endotoxic shock	At 400 mg/kg: PWL restored; ↑ 67% survival rate (72 h) by ↓ TNF-α (500 to 325 pg/mL) and IL-6 (80 to 58 ng/mL).	[50]
Brazil	Turbo-extraction in water and acetone: H_2_O 70% (*v*/*v*) (20 min)	Swiss mice	Carrageenan-induced peritonitis, acetic acid-induced abdominal writhing and hot plate test	At 50mg/kg: number of leukocyte migration into the peritoneal cavity H_2_O < H_2_O -acetone extract. No central analgesic activity. Peripheral analgesic activity: ↓ number writhing response (from 50 to 15 count).	[19]
India	Maceration in EtOH (7 days)	Wistar rats	Acetic acid-induced writhing	At 2 mg/kg ↓ 66% number writhing response (from 67 to 54 count). Comparable to diclofenac sodium (75%).	[107]
India	Distillation with MeOH and H_2_O	Wistar rats	Acetic acid-induced writhing and hot plate test	At 10 and 30 mg/kg ↓ responses time (at 9.4 and 10.6 s) compared to the analgesic drug Pentazocine (14 s).	[108]

Hemoglobin (Hb); interleukin-6 (IL-6); lipopolysaccharide (LPS); mean concentration hemoglobin count (MCHC); mean corpuscular volume (MCV); packed cell volume (PCV); paw withdrawal latency (PWL); red-blood cell counts (RBCC); tumor necrosis factor alpha (TNF-α); white-blood cell (WBC); ↑ increases the affect; ↓ decreases the effect.

**Table 8:** Endocrine and metabolic in vivo assays with guava leaves.

Origin	Subject	Treatment	Main Results	Ref.
Nigeria	Rabbits	High-cholesterol diet	At 250 mg/kg: ↓ TC (15%); ↑ HDL (69%); ↓ LDL (74%); ↓ hyperglycemia 43%.	[109]
Brazil	Wistar rats	High-cholesterol diet	At 369.89 mg phenolic compound in the extract/g: ↓ TC (29–35%), TG (59–73%); ↑ HDL (46%); ↓ VLDL+LDL; ↓ enzyme activity (SOD (6.2 to 5.7 U/mg protein), GP (4.6 to 2.3 µmol/g protein).	[110]
Korea	*Lepr^db^/Lepr^db^* juvenile and adult mice	Diabetes spontaneous mutation	At 10 mg/kg: 87% inhibition PTP1B; ↓ glucose levels 31% and 42% respectively.	[111]
Iran	Wistar rat	Streptozotocin-induced diabetes	At 1mg/L: ↓ Ca/Mg ratio (18 to 12), glucose level, TG (100 to 65 mg/dL), TC (68 to 48 mg/dL), ↑ HDL (18 to 40 mg/dL), ↓ LDL, and VLDL to normal levels; ↓ alteration in vascular reactivity (110 to 50 mmHg).	[112]
Taiwan	Sprague-Dawley rats	Low-dose streptozotocin and nicotinamide-induced diabetes	At 400 mg/kg: ↓ blood glucose level (230 to 140 mg/dL); ↑ plasma insulin level and glucose utilization (normal levels); ↑ enzyme activity (hepatic hexokinase (8 to 11 U/mg protein), phosphofructokinase (18 to 25 U/mg protein) and glucose-6-phosphate dehydrogenase (11 to 25 U/mg protein).	[113]
India	Sprague-Dawley rats	Streptozotocin-induced diabetes	At 100 mg/kg: ↓ blood glucose level (4 to 1 mg/mL) and lipid peroxidation (2 to 1 mmol/100 g tissue); ↑ enzyme activity (CAT (6 to 10 × 10^3^ U/mg protein), SOD (6 to 10 U/mg protein), GPx (0.4 to 0.6 U/mg protein), GRd (0.1 to 0.3 U/mg protein).	[55]
Nigeria	Albino rats	Alloxan-induced diabetes	At 200 mg/kg: ↑ average weight (99 to 209g); ↓ blood glucose level (15 to 8 mmol/L); ↓ alanine aminotransferase activity (32 to 24 U/L).	[114]
India	Albino rats	Alloxan-induced diabetes	At 500 mg/kg: ↓ blood glucose level, TC (231 to 163 mg/dL), TG (133 to 69 mg/dL), LDL (186 to 126 mg/dL), VLDL (26 to 13 mg/dL); ↑ HDL (18 to 23 mg/dL).	[115]
Nigeria	Wister rats	-	At 150 mg/kg: ↑ ALP (300, 175and 650 IU), AST (500, 400, 450 IU), ALT (1200, 1200, 1800 IU), ACP (750, 650, 900 IU) activity in the kidney, liver, and serum, respectively.	[116]
Nigeria	Mice	-	At 49.3 mg/mL: ↑ AST (93 to 126 iµ/L), ALT (30 to 35 iµ/L), ALP (57 to 66 iµ/L), conjugate bilirubin (0.2 to 0.3 mg/dL) and creatinine (0.9 to 1.2 mg/dL).	[106]
Nigeria	Albino rats	-	At 150 mg/kg: ↑ serum urea (2.9 to 6 mmol/L) and creatinine (2.7 to 4 mmol/L); ↓ concentration of serum Na^+^ (122 to 99 mmol/L).	[117]

acid phosphatase (ACP); alanine aminotransferase (ALT); alkaline phosphatase (ALP); aspartate aminotransferase (AST); catalase (CAT); glutathione peroxidase (GPx); glutathione reductase (GRd); high-density lipoprotein (HDL) cholesterol; low-density lipoprotein (LDL) cholesterol; protein tyrosine phosphatase 1B (PTP1B); superoxide dismutase enzyme (SOD); total cholesterol (TC); triglycerides (TG); very low-density lipoprotein (VLDL) cholesterol; ↑ increases the affect; ↓ decreases the effect.

**Table 9:** In vivo assays for digestive system related diseases.

Origin	Extraction Method	Subject	Treatment	Main Results	Ref.
India	Extraction with MeOH	Wistar rats	ASP, PL, and EtOH-induced ulcers	At 200 mg/kg: PL-induced ulcers: ↓ 64% ulcer formation (ui = 2.1), ↓ GV (5 to 2 mL), acid secretion (88 to 64 mEq/L/100 g), ↑ pH (2 to 5).Comparable to omeprazole; ASP (↓ 70.5%, ui = 2.5) and EtOH (↓ 70.4%, ui = 8.7)-induced systems.	[124]
Nigeria	Maceration in H_2_O (24 h)	Albino rats	EtOH-induced ulcers	At 1000 mg/kg: ↓ MNL (9.4 to 2) ui (4.7 to 1).	[125]
Nigeria	Maceration with agitation in MeOH (24 h)	Wistar rats	EtOH-induced ulcers	At 1000 mg/kg: ↓ ui (17.7 to 6.3), ↑ protection (64.4%).	[126]
India	Maceration in EtOH:H_2_O 90% (*v*/*v*) (72 h).	Wistar rats	PL and EtOH-induced ulcers	At 200 mg/kg: PL-induced: ↓ ulcer formation (77 to 84%), ui (5 to 1.3), GV (1.4 to 0.5 mL/100g), and acid secretion (28 to 23 mEq/L); ↑ pH (2.0 to 3.4). EtOH-induced: ↓ (63% to 79%, ui = 1.6 to 5.6), and gastric lesions (5.6–1.9).	[127]
South Africa	Maceration in H_2_O (48 h)	Wistar rats and BALB/c mice	Castor oil-induced diarrhea and castor oil-induced enteropooling	At 400 mg/kg: ↑ 83.3% rat protection, ↓ 75% fluid accumulation in rats; ↓ 87.73% transit in rats and 77.2% in mice; ↓ 64.35% of contractions in mice.	[128]
Nigeria	Soxhlet with EtOH:H_2_O 70% (*v*/*v*)	Wistar rats	Castor oil-induced diarrhea	At 80 mg/kg: ↓ 53.03% transit in rats and ↓ 67.70% intestinal contractions.	[129]
Pakistan	Maceration with EtOH	BALB/c mice, rabbit jejunum	Castor oil-induced diarrhea, K^+^-induced motility	At 1 g/kg: ↑ 81.1% mice protection; Spasmolytic effect (0.3–1 mg/mL) ↓ spontaneous contractions EC_50_ = 0.66 mg/mL in rabbits.	[130]
India	Decoction (1 h)	Wistar rats	CCl_4_, PCM, and TAA-induced liver injury	At 500 mg/kg: CCl_4_: ↓ ALT (384 to 17 U/L), AST (642 to 152 U/L), ALP (750 to 489 U/L), and bilirubin (1.6 to 0.3 mg/dL), ↓ control levels; PCM: ↓ ALT (384 to 87 U/L), AST (642 to 179 U/L), ALP (750 to 338 U/L), and bilirubin (1.6 to 0.6 mg/dL); TAA: ↓ ALT (337 to 32 U/L), AST (438 to 237 U/L), and ALP (770 to 479 U/L).	[131]
India	Soxhlet with EtOH	Wistar rats	PCM-induced liver injury	At 400 mg/kg: ↓ SGOT (475 to 370), SGPT (158 to 128), ALP (814 to 729), and bilirubin (0.7 to 0.6); ↑ total protein (5.15 to 5.6), albumin (2.6 to 3.1), and GLO (2.1 to 2.4). Histopathological observations: less diffuse granular degeneration and mild periportal lymphocytic infiltration.	[132]
India	Decoction (1 h)	Wistar rats	Acetaminophen-induced liver injury	At 500 mg/kg: ↓ AST (121 to 77 IU/L), ALT (80 to 57 IU/L), ALP (115 to 67 IU/L), and total bilirubin (4 to 2 mg/dL). Restored: total protein (5 to 7 g/dL), LPO (7 to 2 nmol/mg protein), GPx (13 to 19 µmol/mg protein), GSH (15 to 23 µmol/mg protein), CAT (14 to 24 µmol/mg protein), and SOD (48 to 63 µmol/mg protein). Histopathological observations: normal lobular structure.	[133]
Egypt	Maceration with agitation in EtOH:H_2_O 70% (*v*/*v*) (24 h)	Albino rats	CCl_4_-induced liver injury	At 500 mg/kg: ↓ ALT (94 to 55 U/mL), AST (199 to 82 U/mL), GGT (71 to 23 U/mL), lysosomal enzymes (50%), and LPO (7 to 3 nmol/mg protein); ↑ SOD (15 to 39 U/mg protein), CAT (5 to 15 µg/mg protein), GSH (6 to 8 µg/mg protein), GST (13 to 25 mM/min/mg protein), total protein (48 to 58 g/L), albumin (29 to 38 g/L), GLO (19 to 21 g/L).	[134]
Egypt	Decoction (1 h)	Wistar rats	PCM-induced liver injury	↓ AST (342 to 156 U/L), ALT (359 to 80 U/L), ALP (288 to 263 U/L), LDH (207 to 143 U/L), GGT (11 to 7 U/L), and total bilirubin (0.3 to 0.2 mg/dL). Restored SOD (13 to 24 U/g) and CAT (5 to 17 U/g).	[135]

Alkaline phosphatase (ALP); Alanine aminotransferase (ALT); aspirin (ASP); aspartate aminotransferase(AST); catalase (CAT); carbon tetrachloride (CCl_4_); ethanol (EtOH); gamma glutamyl transferase (GGT); gastric volume (GV); globulin (GLO); glutathione (GSH); glutathione peroxidase (GPx); glutathione S-transferase (GST); lactate dehydrogenase (LDH); lipid peroxidation (LPO); mean number lesions (MNL); paracetamol (PCM); pyloric ligation (PL), Serum glutamic oxaloacetic transaminase (SGOT); Serum glutamic pyruvic transaminase (SGPT), superoxide dismutase (SOD); thioacetamide (TAA); ulcer index (ui); ↑ increases the affect; ↓ decreases the effect.
